# *Flavobacterium hungaricum* sp. nov. a novel soil inhabitant, cellulolytic bacterium isolated from plough field

**DOI:** 10.1007/s00203-022-02905-x

**Published:** 2022-05-06

**Authors:** Rózsa Máté, József Kutasi, Ildikó Bata-Vidács, Judit Kosztik, József Kukolya, Erika Tóth, Károly Bóka, András Táncsics, Gábor Kovács, István Nagy, Ákos Tóth

**Affiliations:** 1 BioFil Microbiological, Biotechnological and Biochemical Ltd, Budapest, Hungary; 2 Research Group for Food Biotechnology, Institute of Food Science and Technology, Hungarian University of Agriculture and Life Sciences, Budapest, Hungary; 3 Department of Microbiology, Eötvös Loránd University, Budapest, Hungary; 4 Department of Plant Anatomy, Eötvös Loránd University, Budapest, Hungary; 5 Department of Molecular Ecology, Institute of Aquaculture and Environmental Safety, Hungarian University of Agriculture and Life Sciences, Gödöllő, Hungary; 6 SeqOmics Biotechnology Ltd., Mórahalom, Hungary; 7 Department of Molecular Genetics,, Teaching Hospital Markusovszky, Szombathely, Hungary; 8 Institute of Biochemistry, Biological Research Centre, Eötvös Lorand Research Network, Szeged, Hungary

**Keywords:** *Bacteroidetes*, *Flavobacterium*, New taxa, Cellulolytic, glycoside hydrolase

## Abstract

A Gram-negative bacterial strain, named Kb82, was isolated from agricultural soil and a polyphasic approach was used for characterisation and to determine its taxonomic position. Based on 16S rRNA gene sequence analysis, the highest similarity was found with *Flavobacterium artemisiae* SYP-B1015 (98.2%). The highest ANI (83.3%) and dDDH (26.5%) values were found with *Flavobacterium ginsenosidimutans* THG 01 and *Flavobacterium fluviale* HYN0086^T^, respectively. The isolate is aerobic with rod-shaped cells, positive for catalase and negative for oxidase tests. The DNA G+C content is 34.7 mol%. The only isoprenoid quinone is menaquinone 6 (MK-6). The major fatty acids are iso-C_15:0_, summed feature 3 (C_16:1_ *ω7c*/C_16:1_ *ω6c*) and iso-C_17:0_ 3OH. The major polar lipid is phosphatidylethanolamine. On the bases of phenotypic characteristics and analysis of 16S rRNA gene sequences, it is concluded that strain Kb82^T^ represents a novel species in the *Flavobacterium* genus, for which the name *Flavobacterium hungaricum* sp. nov. is proposed. The type strain of the species is strain Kb82^T^ (= LMG 31576^T^ = NCAIM B.02635^T^).

**Supplementary Information:**

The online version contains supplementary material available at 10.1007/s00203-022-02905-x.

## Introduction

The genus *Flavobacterium* was described by Bergey et al. (1923) emended by Bernardet et al. (1996), Dong et al. (2013), Kang et al. (2013) and Kuo et al. (2013). The genus belongs to the family *Flavobacteriaceae*, order *Flavobacteriales*, class *Flavobacteriia*, phylum ‘*Bacteroidetes*’.

This group of Bacteria is very diverse and strains have been isolated from a wide variety of habitats. The genus includes 257 validly published species with correct names (https://lpsn.dsmz.de/ February 2022) (Parte et al. 2020) at the time of writing, the type species is *Flavobacterium aquatile* (Frankland and Frankland 1889). Culture-based and culture-independent studies indicate that *Flavobacteria* are one of the most abundant group in soil, especially in the rhizosphere. Members of the *Flavobacterium* are often associated with the capacity to degrade complex organic compounds in soil. Many recent studies suggest that these bacteria have a plant growth-promoting properties, particularly in the early and intermediate growth stages (Kolton et al. 2016). Organisms that are able to break down biopolymers play a pivotal role in the turnover of various organic matter in the soil. Lignocellulose is a complex biopolymer consisting of cellulose, hemi cellulose and lignin. In the background of chemical and biological resistance stand chemical complexity and an arranged structure. The phenotypic, chemotaxonomic and genotypic properties indicate that strain Kb82^T^ represents a novel species within the genus *Flavobacterium*, for which the name *Flavobacterium hungaricum* sp. nov. is proposed. The type strain of the species is strain Kb82^T^ (= LMG 31576^T^ = NCAIM B.02635^T^).

## Materials and methods

### Isolation and cultivation

Strain Kb82^T^ was isolated in the Great Hungarian Plain, from an agricultural field, after the maize was harvested. The soil with a pH moderately alkaline was fertilised. For the study, soil particles were homogenised by vortexing and serial dilutions were prepared with peptone water (1 g peptone, 9 g NaCl, in 1000 ml dH_2_O). 100–100 µl of the third to the fifth member of the dilution series was spread onto Distillers Dried Grains with Solubles (DDGS) containing agar (1 g NaNO_3_; 1 g K_2_HPO_4_; 3 g NaCl; 0.5 g MgCl_2_; 0.5 g yeast extract; 0.5 g peptone; 3 g DDGS; 25 g agar; 1000 ml dH_2_O). The plated were incubated at 10 °C for 5 days. Single colonies were taken from the plates and purified on the same medium. All similar phenotypes’ 16S rRNA gene were sequenced. The isolate was maintained on LB medium (DSM medium No. 381, www.dsmz.de) at 28 °C and pH 7.5. but the novel strain also grows well on TSA, nutrient, R2A and minimal media with xylan, mannan and carboxymethyl cellulose (CMC) as the sole carbon source (1 g NaNO_3_; 1 g K_2_HPO_4_; 3 g NaCl; 0.5 g MgCl_2_; 4 g xylan/mannan/CMC; 25 g agar; 1000 ml dH_2_O).

### Physiology and chemotaxonomy

For the chemical and molecular studies, biomass was prepared by cultivation in shaker flasks in LB medium at 28 °C for 32 h. The colony morphology of the strain was studied on LB agar medium by directly observing single colonies. Presence of flexirubin type pigment (by 20% KOH), production of brown diffusible pigment on l-tyrosine agar, absorption of Congo red degradation of agar, casein, chitin, pectin, DNA, l-tyrosine, production of H_2_S and gliding motility (by the hanging-drop technique) were estimated according to the minimal standards for describing novel taxa in the family *Flavobacteriaceae* (Bernardet et al. 2002) and Barrow and Feltham (2004). Cell morphology of the strain was observed by electron microscopy. Gram reaction was studied with the non-staining method of Buck (1982). Oxidase activity was determined with OXI oxidase test strip (Diagnostics s.r.o.). Catalase production was shown according to Barrow and Feltham (2004). The effects of different temperatures (from 4 to 50 °C) on the growth of the bacterium, NaCl (0–4% w/v) and pH (pH 4–10, using increments of 0.5 pH units) tolerances were determined in LB medium. API 50 CH, API 20 NE and API ZYM kits (BioMérieux) were used according to the manufacturer’s instructions for determining acid production from different carbon sources, the assimilation of different substrates and the enzymatic activities of the strain. The API 50 CH and 20 NE tests were read after 24–48 h incubation at 28 °C. Growth under anaerobic and microaerophilic conditions was checked on LB medium with the help of Anaerocult A and C systems (Merck). The physiological characteristics were compared to the closely related *Flavobacterium compostarboris* JCM 16527^T^ by side-by-side analysis.

Chemotaxonomic traits were analysed by DSMZ Identification Service (DSMZ, Braunschweig, Germany). Active growing cultures of the strain on LB agar were used in the analysis of the fatty acid profiles of the strain. According to the DSMZ Identification Service, fatty acid methyl esters (FAMEs) were obtained following the methods of Miller (1982) and Kuykendall et al. (1988). Gas chromatography was used for the separation of FAMEs, which were detected by a flame ionisation detector using the Sherlock Microbial Identification System (MIS) (MIDI, Microbial ID, Newark, DE 19711 U.S.A.). FAMEs were identified by using the TSBA6 6.10 database of the Microbial Identification System. GC/MS was used for the identification of summed feature components thereafter.

Respiratory quinones were extracted from freeze-dried material and silica-based solid phase extraction method was used for purification. The purified samples were further analysed by HPLC and UHPLC-ESI-qTOF systems (Tindall 1990a, b; dsmz.de). Polar lipids were determined based on the methods of Tindall et al. (1990a, b; Tindall et al. 2007; dsmz.de).

### Genome features

DNA was extracted from Kb82^T^ liquid culture grown in LB medium. Genomic DNA isolation and 16S rRNA gene amplification were performed according to Tóth et al. (2017). Sequencing of the genome of the strain was done with Illumina MiSeq sequencing technology according to Szuroczki et al. (2019). Genome assembly was performed by SPAdes v. 3.9.1; CLC NGS Cell v. 11.0. Genome completeness and contamination values were studied by TypeMet tool of MiGA server (http://microbial-genomes.org/) (Rodriguez-R et al. 2018). ANI and digital DNA–DNA hybridisation (dDDH; identities/HSP length) values were determined using the OrthoANI algorithm (www. ezbiocloud. net/ tools/ ani) (Yoon et al. 2017) and Genome-to-Genome Distance Calculator service of DSMZ (http:// ggdc. dsmz. de/) (Meier-Kolthoff et al. 2013). Annotation of the genome was performed by NCBI Prokaryotic Genome Annotation Pipeline v4.4 with Best-placed reference protein set and GeneMarkS + methods (Tatusova et al. 2016; O’Leary et al. 2016) and Rapid Annotation using Subsystem Technology server v. 2.0 (RAST; https://rast.nmpdr.org) (Aziz et al. 2008).

To identify the secondary metabolite biosynthesis gene clusters, the anti-SMASH server was used (Blin et al. 2019).

## Phylogeny

The partial 16S rRNA gene sequence of the strain was compared with the EzTaxon EzBioCloud Database (http://www.ezbiocloud.net/taxonomy) (Kim et al. 2012a, b) for an approximate phylogenetic affiliation. After Sanger sequencing of the 16S rRNA gene, a genome sequencing project of Kb82^T^ was carried out, which revealed that there is only one 16S rRNA gene copy in the genome. Phylogenetic trees were built by using the neighbor-joining (Saitou and Nei 1987) and maximum-likelihood (Felsenstein 1981) methods with Kimura’s two-parameter calculation model and the maximum-parsimony algorithm (Kimura 1980) using MEGA version 10.0 (Kumar et al. 2018). Tree topologies and distances were evaluated by bootstrap analysis based on 1000 replicates. For phylogenomic studies TYGS (https://tygs.dsmz.de/) (Meier-Kolthoff and Göker 2019), MiGA (http://microbial-genomes.org/) (Rodriguez-R et al. 2018) and GGDC (http://ggdc.dsmz.de/) (Meier-Kolthoff et al. 2013) webservers were used.

## Results and discussion

### Phenotypic and biochemical characterisation

Distinctive physiological and biochemical characteristics of the isolate are given in Table 1. List of all negative traits from API tests is presented in Online resource 1. The other morphological and physiological characteristics are listed in the species description.

**Table 1:** Differential characteristics of Kb82^T^ (1) and the closely related *Flavobacterium compostarboris* JCM 16527^T^ (2) *Flavobacterium artemisiae* SYP-B1015^T^ (3), *Flavobacterium crocinum* HYN0056^T^ (4), *Flavobacterium quisquiliarum* EA-12^T^ (5)* Data are from Kim et al. (2012a, b), Zhao et al. (2018), Baek et al. (2018) and Zhang et al. (2017)

	1	2	3*	4*	5*
Isolation source	Soil	Compost	Rhizosphere	Freshwater	Activated sludge
Temperature range for growth (°C) (optimum)	10–30 (25)	15–30 (30)	5–34 (24–30)	4–40 (30)	15–37 (25)
pH range for growth (optimum)	5.5–8.5 (7.0)	5.5–8.0 (6.5)	5.0–8.0 (7.0)	5.0–11.5 (7.5)	5.0–9.0 (7.0)
Indole production	−	−	+	−	−
Hydrolysis of					
Esculin	+	+	−	+	+
CM-cellulose	+	+	−	−	−
Starch	+	+	+	+	−
Acid production from					
d-trehalose	−	+	nd	nd	nd
l-arabinose	−	−	+	nd	+
Maltose	+	+	−	nd	+
Potassium 5-ketogluconate	+	−	nd	nd	nd
Activity of					
N-acetyl-β-glucosaminidase	−	+	nd	+	+
oxidase	−	−	−	+	+
DNA G + C content (mol%)	34.7	33.6*	33.5	34.0	36.1

### Chemotaxonomic characteristics

The predominant cellular fatty acids of the strain were found to be iso-C_15:0_ (32.6%), summed feature 3 (C_16:1_ *ω*7*c*/C_16:1_ *ω*6*c*, 14.6%) and iso-C_17:0_ 3OH (11.6%). The fatty acid profile is similar to that of related strains, in accordance with the description of *Flavobacterium* genus (Kang et al. 2013), though the ratios of the different components are different. The complete fatty acid composition is shown in Online resource 2. The only respiratory quinone of Kb82^T^ is menaquinone-6 (MK-6). Strain Kb82^T^ exhibits a complex polar lipid profile consisting of one phosphatidylethanolamine (PE) as the dominant element, two aminolipids (AL), three phospholipids (PL), one aminoglycolipid (GNL) and seven uncharacterised lipids (L) (Online resource 3).

### Whole-genome sequence analysis

The completeness and contamination values of the genome are 97.2 and 1.9%, respectively. Other quality labels of genome sequencing and assembly are as follows: 155-fold genome coverage, contig N50 = 1,261,348 bp, number of contigs are 12. The genome size and G + C content of Kb82^T^ are 5,872,517 bp and 34.7 mol%, respectively. According to the annotation, there are 5178 genes, 5088 CDSs and 90 RNA genes in the genome. The coding density is 87.0%. The genomic traits of the strain and related type strains are summarised in Table 2.

**Table 2:** Genome sequencing summaries and general characteristics of strain Kb82^T^ and closely related type strains, and their pair-wise average nucleotide identity (ANI) and digital DNA–DNA hybridization (DDH) values

Characteristic	Strains	1	2	3	4
Number of contigs		12	1	1	82
Size (base)		5,872,517	4,839,571	5,877,431	5,483,841
DNA G+C content (mol%)		34.7	33.9	34.0	33.5
Total number of genes		5178	4183	4992	4694
Number of protein-coding genes		5033	4065	4879	4591
Number of tRNA genes		65	60	63	52
Digital DDH value (%, below the diagonal)		ANI value (%, above the diagonal)			
	1	100	82.5	81.5	82.1
	2	26.5	100	84.2	84.3
	3	25.1	28.2	100	84.7
	4	25.8	28.6	29.4	100

Strains: 1. *Flavobacterium hungaricum* Kb82^T^ (GCA_015182285.1), 2. *Flavobacterium fluviale* (GCA_003312915), 3. *Flavobacterium crocinum* HYN0056^T^ (GCA_003122385.1), 4. *Flavobacterium ginsenosidimutans* (GCA_003254625.1)

The RAST analysis revealed the presence of 277 subsystems, the subsystem coverage was 17% (Online resource 4). The genome of Kb82^T^ contains 10 putative biosynthetic gene clusters (Non-ribosomal peptide synthetase, Type I polyketide synthase, Type III polyketide synthase, betalactone, arylpolyene, resorcinol, proteusin, siderophore, terpene, Class I lanthipeptide clusters like nisin) in 9 genomic regions. Based on the RAST and the anti-SMASH server the strain encodes genes required for siderophore production. The production of siderophores can promote plants health by the suppression of pathogens (Rana et al. 2020).

Using the genome annotation and the Pfam database (Mistry et al. 2020; http://pfam.xfam.org/), several glycoside hydrolase (GH) genes in various GH families were found, which indicates that the strain Kb82^T^ specialises in the breakdown of complex plant-associated carbohydrates. Genome sequence analysis also revealed three genes from glycoside hydrolase families GH78 (GenBank accession: MBE8726248, MBE8726269 and MBE8726274) and one rhamnogalacturonan acetylesterase gene (GenBank accession: MBE8726158) which may play a role in rhamnogalacturonan utilisation. Because this polymer is present only in terrestrial plants, these genes can only be found in the genomes of the terrestrial clade (Kolton et al. 2013). Cellulose degradation was proven by Congo red staining and the strain is able to grow on a minimal medium with polysaccharides as the sole carbon source. In the genome of the strain enzyme genes have been identified that may play a role in the breakdown of lignocellulose (http://www.cazy.org/) (Lombard et al. 2014). We identified 100 GH genes in 32 GH families (Online resource 5).

As a result of genome analysis, several genes involved in flavobacterial gliding motility (*GldN, GldK, GldL, GldM, GldI, GldA, GldE, GldD, GldJ, GldB, GldC, GldH, GldG, GldF, SprA, SprE, SprF, SprT, ChiA, RemB*) have been identified in Kb82^T^ genome (McBride and Nakane 2015; Penttinen et al. 2018). A subset of these genes has been found to form a protein translocation system called type IX secretion system (T9SS) restricted to ‘*Bacteroidetes*’. T9SS has an important role in the secretion of gliding motility adhesins. Several studies indicate the significant role of *Flavobacterium* strains in soil and especially in the rhizosphere. In such highly competitive ecosystems, the large numbers of glycoside hydrolase genes and the special gliding mobility may help in the successful colonisation of niches (Kolton et al. 2016).

### Phylogenetic analysis

**Fig. 1:**
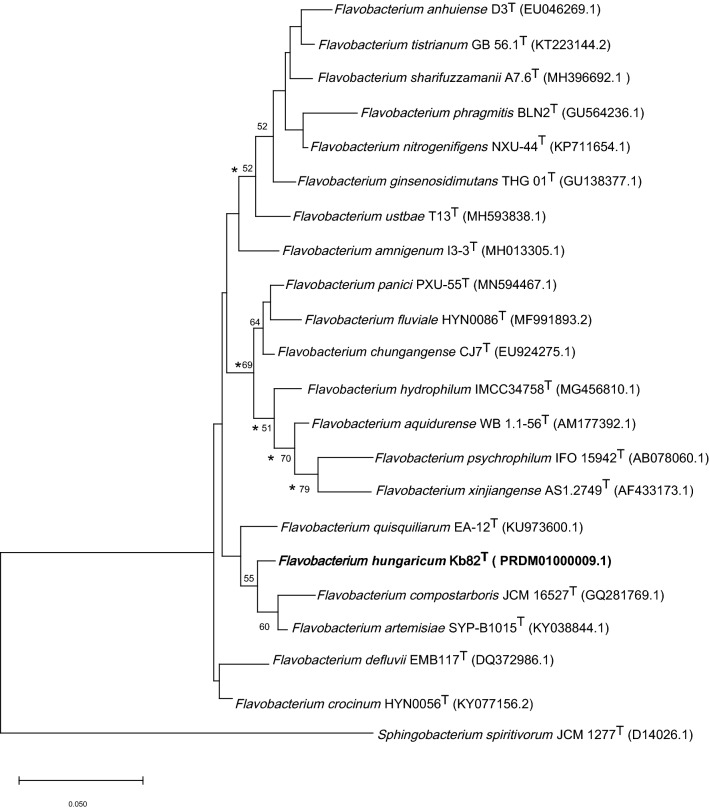
Maximum-likelihood tree based on 16S rRNA gene sequences showing the phylogenetic relationships between strain Kb82^T^ and related taxa. Bootstrap values (>50%) are shown as percentages of 1000 replicates. Branches with lower bootstrap values than 50% are uncertain. Branches signed with an asterisk occurred with every tree-making algorithm used in the study. Bar, 0.05 substitution per nucleotide position

According to the comparisons with the complete 16S rRNA gene sequences in the EzTaxon database, the highest level of sequence similarity occurred with *Flavobacterium artemisiae* SYP-B1015^T^ (98.2%) (Zhao et al. 2018), followed by *Flavobacterium crocinum* HYN0056^T^ (97.4%) (Baek et al. 2018) and *Flavobacterium compostarboris* 15C3^T^ (97.3%) (Kim et al. 2012a, b). The 16S rRNA gene based phylogeny tree suggests that strain Kb82^T^ forms a distinct phyletic lineage within *Flavobacterium* genus (Fig. 1).

According to genome-based analysis, the closely related taxons found by MiGA are *Flavobacterium ginsenosidimutans* THG01^**T**^ (Yang et al 2011) (GenBank assembly accession: GCA_003254625) (83.3% ANI) and *Flavobacterium sharifuzzamanii* A7.6^ T^ (Debnath et al 2019) (GenBank assembly accession: GCA_003254585**)** (83.2% ANI). The p-value of taxonomic novelty at the species level is 0.00269.

The highest dDDH value (identities/HSP length) between Kb82^T^ and related strains was found with *Flavobacterium fluviale* HYN0086^T^ (Baek et al 2020) (GenBank assembly accession: GCA_003312915) (26.5%). Whole genome-based tree generated by TYGS also confirmed the taxonomic position of Kb82^T^ within *Flavobacterium* genus as a novel species (Online resource 6).

According to the 16S rRNA based and whole genome based phylogenetic analyses, Kb82^T^ represents a novel species in genus *Flavobacterium*. The generally accepted species boundary for 16S rRNA gene similarity, ANI and dDDH values are 98.7, 95–96 and 70%, respectively (Chun et al. 2018). Obtained values for Kb82^T^ (98.2% for 16S rRNA gene similarity, 83.3% for ANI and 26.5% for dDDH) are all lower, confirming the results of phylogenetic treeing.

In conclusion, the phenotypic, biochemical, chemotaxonomic and phylogenetic information of strain Kb82^T^ support its classification as a novel species of *Flavobacterium*, for which the name *Flavobacterium hungaricum* sp. nov. is proposed.

## Description of *Flavobacterium hungaricum* sp. nov.

*Flavobacterium hungaricum* (hun.ga'ri.cum. M.L. neut. adj. hungaricum of or belonging to Hungary, where the type strain was isolated).

Grows well on LB, TSA, nutrient and R2A plates. Colonies are circular, non-mucoid, smooth and have orange pigmentation on LB after 72 h incubation. Flexirubin-type pigment are present. Congo red is not absorbed by colonies. Cells are motile by gliding, strictly aerobic, Gram-reaction-negative, oxidase negative and catalase-positive straight rods. Cells are 0.5 µm in width and 1.5–2.0 µm in length. Individual cells form filaments. It grows at 10–30 °C (optimum, 25 °C), pH 5.5–9.0 (optimum, 7.0) and at NaCl concentrations of 0.0–2.0 w/v % (optimum, 0 w/v %). Able to degrade cellulose, casein, l-tyrosine and esculin, Positive for H_2_S production and acid production from d-xylose, d-galactose, d-glucose, d-fructose, d-mannose, N-acetyl-glucosamine, amygdalin, arbutin, esculin, salicin, d-cellobiose, d-maltose, d-lactose, starch, glycogen, gentobiose, l-fucose, potassium 5-ketogluconate, assimilation of d-glucose, l-arabinose, d-mannose, N-acetyl-glucosamine, d-maltose and β-galactosidase, alkaline phosphatase, leucine arylamidase, valin arylamidase, acid phosphatase naphthol-AS-BI-phosphohydrolase, β-glucosidase activity. The major fatty acids are iso-C_15:0_, summed feature 3 (C_16:1_ *ω*7*c*/C_16:1_ *ω*6*c*) and iso-C_17:0_ 3OH. The only respiratory quinone is MK-6. The major polar lipid is phosphatidylethanolamine. The DNA G + C content is 34.7 mol%.

The type strain is Kb82^T^ (= LMG 31576^T^ = NCAIM B.02635^T^) isolated from an agricultural field in the Great Hungarian Plain.

## Supplementary Information

Below is the link to the electronic supplementary material.

Supplementary file1 (DOC 40 KB)

Supplementary file2 (DOCX 18 KB)

Supplementary file3 (DOCX 150 KB)

Supplementary file4 (DOCX 57 KB)

Supplementary file5 (DOC 49 KB)

Supplementary file6 (DOC 49 KB)

## Data Availability

The GenBank accession numbers for the 16S rRNA gene sequence and the whole genome of *Flavobacterium hungaricum* strain Kb82^T^ are MF471352 and PRDM01000000, respectively. The type strain of the species is deposited in Belgian Coordinated Collections of Microorganisms (LMG 31576^ T^) and Hungarian National Collection of Agricultural and Industrial Microorganisms (NCAIM B.02635^ T^).

## Abbreviations

ANI: Average Nucleotide Identity
dDDH: Digital DNA–DNA hybridisation
DDGS: Distillers Dried Grains with Solubles
DSMZ: Deutsche Sammlung von Mikroorganismen und Zellkulturen (German Collection of Microorganisms and Cell Cultures)
GGDC: Genome-to-Genome Distance Calculator
GNL: Aminoglycolipid
L: Lipid
LB agar: Luria–Bertani agar
MiGA: Microbial Genomes Atlas
MLSA: MultiLocus Sequence Analysis
PE: Phosphatidylethanolamine
PGL: Phosphoglycolipid
PL: Phospholipid
RAST: Rapid Annotation using Subsystem Technology
TYGS: Type Strain Genome Server
