# Supplementary material for: Flavobacterium hungaricum sp. nov. a novel soil inhabitant, cellulolytic bacterium isolated from plough field
Source: Arch Microbiol. 2022 May 6;204(6):301. doi: 10.1007/s00203-022-02905-x (PMC9076710; doi:10.1007/s00203-022-02905-x)
Supplement: Supplementary file 1 — Supplementary file1 (DOC 40 KB) [file 203_2022_2905_MOESM1_ESM.doc]

***Flavobacterium hungaricum*** **sp. nov. a novel soil inhabitant, cellulolytic bacterium isolated from plough field**

Archives of Microbiology

Rózsa Máté^1^, József Kutasi^1^, Ildikó Bata-Vidács^2^, Judit Kosztik^2^, József Kukolya^2^, Erika Tóth^3^, Károly Bóka^4^, András Táncsics^5^, Gábor Kovács^6,7^, István Nagy^6,8^, Ákos Tóth^2, *^

^*^Correspondence: Ákos Tóth; affiliation: Research Group for Food Biotechnology, Institute of Food Science and Technology, Hungarian University of Agriculture and Life Sciences, Budapest, Hungary; e-mail address: Toth.Akos.Gergely@uni-mate.hu

| **Supplementary table 1.** List of negative traits from biochemical tests  esterase (C4), esterase lipase (C8), lipase (C14), cystine-arylamidase, trypsine, α-chymotrypsin, α-galactosidase, β-galactosidase, β-glucuronidase, α-glucosidase, N-acetyl-β- glucosaminidase, α-mannosidase, α-fucosidase, indole production, D-glucose fermentation, arginine dihydrolase, urease, degradation of agar, gelatin, chitin, DNA and pectin, assimilation of D-mannitol, potassium gluconate, capric acid, adipic acid, malic acid, phenylacetic acid and trisodium citrate, acid production from glycerol, erythritol, D-arabinose, L-arabinose, D-ribose, L-xylose, D-adonitol, methyl β-D-xylopyranoside, L-sorbose, L-rhamnose, dulcitol, inositol, D-mannitol, D-sorbitol, methyl α-D-mannopyranoside, methyl α-D-glucopyranoside, D-melibiose, D-saccharose, D-trehalose, inuline, D-melezitose, D-raffinose, xylitol, D-turanose, D-lyxose, D-tagatose, D-fucose, D-arabitol, L-arabitol, K-gluconate, 2-ceto-gluconate, diffusible pigment production on L-tyrosine agar, growth on marine agar |  |  |
| --- | --- | --- |
|  |  |  |
|  |  |  |
|  |  |  |
|  |  |  |
|  |  |  |
|  |  |  |
|  |  |  |
|  |  |  |
|  |  |  |
|  |  |  |
|  |  |  |
|  |  |  |
|  |  |  |
|  |  |  |
