# Supplementary material for: Flavobacterium hungaricum sp. nov. a novel soil inhabitant, cellulolytic bacterium isolated from plough field
Source: Arch Microbiol. 2022 May 6;204(6):301. doi: 10.1007/s00203-022-02905-x (PMC9076710; doi:10.1007/s00203-022-02905-x)
Supplement: Supplementary file 2 — Supplementary file2 (DOCX 18 KB) [file 203_2022_2905_MOESM2_ESM.docx]

***Flavobacterium hungaricum*** **sp. nov. a novel soil inhabitant, cellulolytic bacterium isolated from plough field**

Archives of Microbiology

Rózsa Máté^1^, József Kutasi^1^, Ildikó Bata-Vidács^2^, Judit Kosztik^2^, József Kukolya^2^, Erika Tóth^3^, Károly Bóka^4^, András Táncsics^5^, Gábor Kovács^6,7^, István Nagy^6,8^, Ákos Tóth^2, *^

^*^Correspondence: Ákos Tóth; affiliation: Research Group for Food Biotechnology, Institute of Food Science and Technology, Hungarian University of Agriculture and Life Sciences, Budapest, Hungary; e-mail address: Toth.Akos.Gergely@uni-mate.hu

**Supplementary table 2.** Cellular fatty acid composition of Kb82^T^ (1), *Flavobacterium compostarboris* JCM 16527^T^ (2), *Flavobacterium artemisiae* SYP-B1015^T^ (3), *Flavobacterium crocinum* HYN0056^T^ (4), *Flavobacterium quisquiliarum* EA-12^T^ (5). * Data are from Kim et al, 2012; Zhao et al, 2018; Baek et al, 2018 and Zhang et al, 2017. tr, trace amount (<1 %); -, not detected

|  | **1** | **2** | **3** | **4** | **5** |
| --- | --- | --- | --- | --- | --- |
| C_13:1_ | - | - | - | 1.2 | - |
| C_14:0_ | - | - | - | 1.2 | 2.1 |
| C_15:0_ 3OH | 1.5 | - | - | 1.5 | - |
| iso-C_15:0_ | 32.6 | 37.1 | 26.0 | 22.3 | 25.0 |
| iso-C_15:1_ G | 2.0 | 4.5 | 3.0 | 5.4 | 4.2 |
| iso-C_15:0_ 3OH | 8.7 | 9.6 | 14.4 | 8.1 | 9.7 |
| anteiso-C_15:0_ | 2.9 | 2.0 | tr | 2.5 | tr |
| C_15:1_ *ω*6*c* | 2.7 | 4.2 | 4.0 | 2.6 | 1.1 |
| C_16:0_ | 4.7 | 2.7 | 1.7 | 6.5 | 5.4 |
| C_16:0_ 3OH | 1.9 | 1.1 | 2.7 | 6.9 | 12.5 |
| C_16:0_ | - | - | tr | 1.0 | - |
| iso-C_16:0_ 3OH | 1.3 | tr | 3.7 | 2.8 | 1.4 |
| iso-C_17:0_ | 1.0 | tr | - | - | - |
| iso-C_17:0_ 3OH | 11.6 | 12.5 | 13.4 | 10.2 | 7.0 |
| C_17:1_ *ω*6*c* | 2.1 | 2.3 | 3.3 | 2.3 | tr |
| C_17:1_ *ω* 8*c* | - | - | - | 1.0 | tr |
| C_17:1_ *ω*9*c* | - | - | 3.4 | - | - |
| summed feature 2 C_12:0_ aldehyde / unknown 10.928 | - | - | - | 1.2 | - |
| summed feature 3 (C_16:1_ *ω*7*c*/ C_16:1_ *ω*6*c*) | 14.6 | 9.0 | 3.6 | 18.2 | 17.6 |
| summed feature 4 (iso-C_17:1_ I/anteiso C_17:1_ B) | - | 2.0 | - | - | - |
| summed feature 5 (iso-C_17:1_ I/DMA C_16:0_) | - | - | - | - | 1.6 |
| summed feature 9 (iso-C_17:1_ *ω*9*c/*C_16:0_ 10-methyl) | 7.4 | 6.8 | - | 1.9 | 1.2 |
| summed feature 10 (C_18:1_*ω7c* /C_18:1_*ω6c*) | - | - | 3.0 | - | - |
