# Supplementary material for: Flavobacterium hungaricum sp. nov. a novel soil inhabitant, cellulolytic bacterium isolated from plough field
Source: Arch Microbiol. 2022 May 6;204(6):301. doi: 10.1007/s00203-022-02905-x (PMC9076710; doi:10.1007/s00203-022-02905-x)

***Flavobacterium hungaricum*** **sp. nov. a novel soil inhabitant, cellulolytic bacterium isolated from plough field**

Archives of Microbiology

Rózsa Máté^1^, József Kutasi^1^, Ildikó Bata-Vidács^2^, Judit Kosztik^2^, József Kukolya^2^, Erika Tóth^3^, Károly Bóka^4^, András Táncsics^5^, Gábor Kovács^6,7^, István Nagy^6,8^, Ákos Tóth^2, *^

^*^Correspondence: Ákos Tóth; affiliation: Research Group for Food Biotechnology, Institute of Food Science and Technology, Hungarian University of Agriculture and Life Sciences, Budapest, Hungary; e-mail address: Toth.Akos.Gergely@uni-mate.hu

**Supplementary figure 2.** Subsystems of strain Kb82^T^ revealed by RAST server version 2.0. Genes for amino acid metabolism, carbohydrate metabolism and cofactors/vitamins/prosthetic groups/pigments metabolism constitute the three largest subsystems.


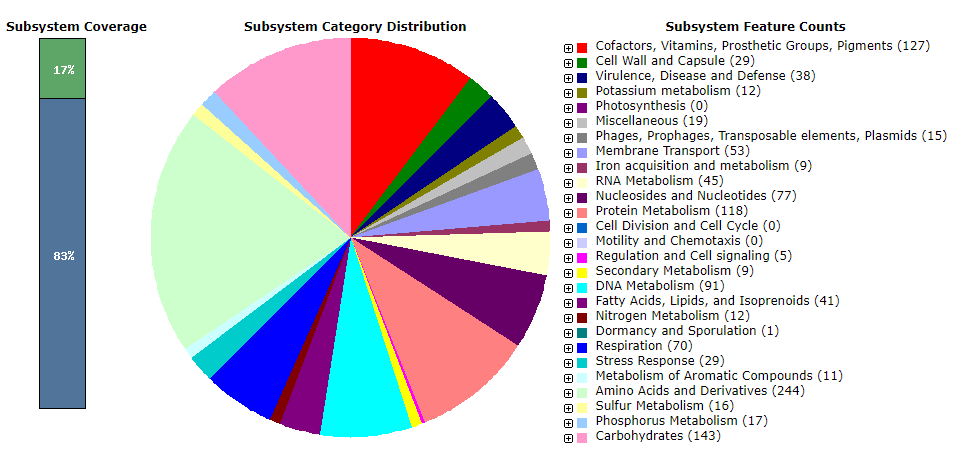

Supplement: Supplementary file 4 — Supplementary file4 (DOCX 57 KB) [file 203_2022_2905_MOESM4_ESM.docx]
