# Supplementary material for: Flavobacterium hungaricum sp. nov. a novel soil inhabitant, cellulolytic bacterium isolated from plough field
Source: Arch Microbiol. 2022 May 6;204(6):301. doi: 10.1007/s00203-022-02905-x (PMC9076710; doi:10.1007/s00203-022-02905-x)
Supplement: Supplementary file 5 — Supplementary file5 (DOC 49 KB) [file 203_2022_2905_MOESM5_ESM.doc]

***Flavobacterium hungaricum*** **sp. nov. a novel soil inhabitant, cellulolytic bacterium isolated from plough field**

Archives of Microbiology

Rózsa Máté^1^, József Kutasi^1^, Ildikó Bata-Vidács^2^, Judit Kosztik^2^, József Kukolya^2^, Erika Tóth^3^, Károly Bóka^4^, András Táncsics^5^, Gábor Kovács^6,7^, István Nagy^6,8^, Ákos Tóth^2, *^

^*^Correspondence: Ákos Tóth; affiliation: Research Group for Food Biotechnology, Institute of Food Science and Technology, Hungarian University of Agriculture and Life Sciences, Budapest, Hungary; e-mail address: Toth.Akos.Gergely@uni-mate.hu

**Supplementary table 3.** Glycoside hydrolase genes found in Kb82^T^ genome, which ones may play a role in plant polysaccharides

| **Glycoside hydrolase family** | **GenBank accession** |
| --- | --- |
| GH 1 | MBE8726894 |
| GH 2 | MBE8724324, MBE8726182, MBE8724461, MBE8726280 |
| GH 3 | MBE8723486, MBE8726298, MBE8724080, MBE8724217, MBE8724490, MBE8725437, MBE8725958, MBE8725960, MBE8727007, MBE8727412 |
| GH 5 | MBE8724322, MBE8727004, MBE8726991 |
| GH 10 | MBE8723323, MBE8723333, MBE8723503 |
| GH 13 | MBE8724027, MBE8724839, MBE8725255, MBE8725254, MBE8725896 |
| GH 16 | MBE8723822, MBE8723823 |
| GH 18 | MBE8726538, MBE8726815 |
| GH 20 | MBE8726294, MBE8726539, MBE8727300 |
| GH 25 | MBE8727803 |
| GH 26 | MBE8728338, MBE8726998, MBE8727003 |
| GH 27 | MBE8724462, MBE8726992 |
| GH 28 | MBE8724457, MBE8724458, MBE8724498, MBE8726147, MBE8726157, MBE8726237, MBE8726266 |
| GH 29 | MBE8726284, MBE8726296 |
| GH 30 | MBE8725432, MBE8725433 |
| GH 31 | MBE8727415 |
| GH 32 | MBE8723349 |
| GH 35 | MBE8726295 |
| GH 37 | MBE8726273 |
| GH 39 | MBE8727413 |
| GH 43 | MBE8723325, MBE8724082, MBE8724323, MBE8724325, MBE8724327, MBE8724463, MBE8724496, MBE8726155, MBE8726267 |
| GH 65 | MBE8725257 |
| GH 67 | MBE8723319 |
| GH 78 | MBE8726248, MBE8726269, MBE8726274 |
| GH 88 | MBE8723797, MBE8724497, MBE8726152, MBE8726153, MBE8726154 |
| GH 92 | MBE8723742, MBE8723743, MBE8724447, MBE8724448, MBE8726288, MBE8726297, MBE8728237 |
| GH 95 | MBE8724460, MBE8726271, MBE8727409 |
| GH 97 | MBE8725043, MBE8725256, MBE8726079, MBE8727005, MBE8727414 |
| GH 115 | MBE8723322 |
| GH 125 | MBE8724414 |
| GH 127 | MBE8726256 |
| GH 130 | MBE8724028, MBE8725782, MBE8726290, MBE8727000 |
| unclassified GH | MBE8724459, MBE8724960, MBE8725044 |
