# Supplementary material for: Flavobacterium hungaricum sp. nov. a novel soil inhabitant, cellulolytic bacterium isolated from plough field
Source: Arch Microbiol. 2022 May 6;204(6):301. doi: 10.1007/s00203-022-02905-x (PMC9076710; doi:10.1007/s00203-022-02905-x)

***Flavobacterium hungaricum*** **sp. nov. a novel soil inhabitant, cellulolytic bacterium isolated from plough field**

Archives of Microbiology

Rózsa Máté^1^, József Kutasi^1^, Ildikó Bata-Vidács^2^, Judit Kosztik^2^, József Kukolya^2^, Erika Tóth^3^, Károly Bóka^4^, András Táncsics^5^, Gábor Kovács^6,7^, István Nagy^6,8^, Ákos Tóth^2, *^

^*^Correspondence: Ákos Tóth; affiliation: Research Group for Food Biotechnology, Institute of Food Science and Technology, Hungarian University of Agriculture and Life Sciences, Budapest, Hungary; e-mail address: Toth.Akos.Gergely@uni-mate.hu

**Supplementary figure 3.** Phylogenomic tree inferred with FastME 2.1.6.1 from GBDP (Genome BLAST Distance Phylogeny approach) distances calculated from genome sequences (Lefort et al. 2015). The branch lengths are scaled in terms of GBDP distance formula *d5* (Farris 1972). The numbers above branches are GBDP pseudo-bootstrap support values > 60 % from 100 replications, with an average branch support of 96.6 %


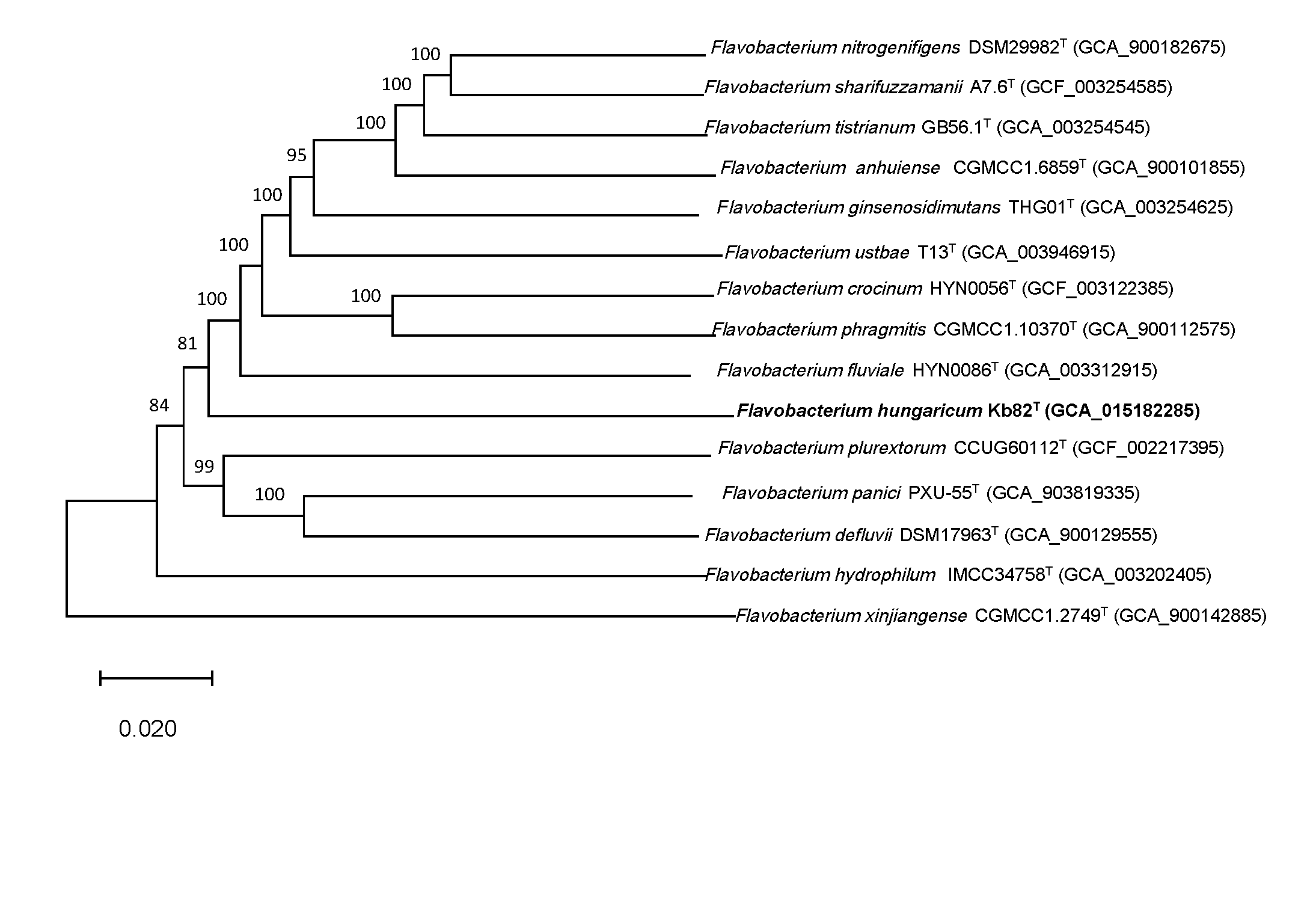

Supplement: Supplementary file 6 — Supplementary file6 (DOC 49 KB) [file 203_2022_2905_MOESM6_ESM.doc]
